# Childhood Tuberculosis: An Under Prioritized Disease in Nepal

**DOI:** 10.31729/jnma.5546

**Published:** 2021-02-28

**Authors:** Rabin Gautam, Gambhir Shrestha, Prabin Phuyal

**Affiliations:** 1 Nepalese Society of Community Medicine, Sanepa, Lalitpur, Nepal; 2 Department of Community Medicine, Maharajgunj Medical Campus, Institute of Medicine, Tribhuvan University, Maharajgunj, Kathmandu, Nepal; 3 BP Koirala Insitute of Health Sciences, Dharan, Sunsari, Nepal

**Keywords:** *case detection*, *childhood tuberculosis*, *national tuberculosis program*

## Abstract

Globally, childhood tuberculosis constitutes up to 10% of overall tuberculosis cases. In Nepal, childhood tuberculosis has remained around 5.5% of overall tuberculosis cases and has remained stagnant over the years. Moreover, our health system is focused on adult tuberculosis. Childhood tuberculosis has recently got its attention both at the national and international levels. National Tuberculosis Program has been a successful program; however, more has to be done to track childhood tuberculosis progress. In this viewpoint, we discuss current initiatives taken by the government and the way forward for case detection and management of childhood tuberculosis in Nepal.

## INTRODUCTION

Tuberculosis (TB) remains a major public health problem worldwide and it is one of the top ten causes of death globally.^1^ At the global level, an estimated 10 million (range, 9.0-11 million) people were diagnosed with new TB cases, among which 1.1 million were children. The Southeast Asia Region (SEARO) accounts for almost half of new TB cases and 50% of TB deaths globally.^2^ TB is also one of the leading causes of Disability-adjusted life years (DALY) in the SEARO region.

## BURDEN OF CHILDHOOD TUBERCULOSIS IN NEPAL

In Nepal (population of 30 million), TB is the 8^th^ common cause of mortality and ranks 12^th^ among the most number of DALYs.^3^ Nepal is a medium burden country for TB with a case notification rate of 109 cases /100,000 population.^4^ Childhood TB accounts for 5.5% of overall TB cases in Nepal, and this has been stagnant over the last few years. Globally childhood TB contributes roughly 10 % of overall TB cases.^5^ This shows that in Nepal considerable gap has been present in the detection of childhood TB. Further, it accounts for 14642 of the total DALY of TB in Nepal.

## SCENARIO OF CHILDHOOD TB IN NEPAL

Childhood TB is usually acquired through infectious adults. Most of the infections are paucibacillary and smear-negative. This is one of the reasons why the global TB control strategy has focused on smear-positive cases. Despite childhood TB not getting enough attention in the past, recently, it has been of focus both at the global and national levels. However, enough priority has still not been set for childhood TB.

The National Tuberculosis Control Program (NTP), looking after TB control in Nepal, has achieved significant success over the last few years. This has largely been attributed to the success of the Directly Observed Treatment Short-course (DOTS) program in Nepal, which started in 1998. The treatment success rate stands at more than 90%. Despite this, there has been stagnant case notification for childhood TB, with only 5.5% of overall TB cases represented by children in 2018/2019.

To address stagnant case notification, the NTP, along with support from partners, have initiated various activities. Some of the activities conducted by the NTP in 2018/2019 were as follows.

1. Development of childhood TB guideline: Nepal developed its first childhood TB guideline in 2018 in collaboration with the Nepal Pediatric Society.^6^ This was the first document to standardize the diagnostic and treatment protocol for the management of childhood TB in Nepal. The overall purpose of the guideline was to help pediatricians, medical officers, and health workers with childhood TB management.
2. Cash incentive for referral of probable childhood TB cases from Health Post/Primary Health Care Center to different hospitals
3. Childhood TB management in major hospitals across Nepal
4. Initiation of Isoniazid Preventive Therapy (IPT) to under 5 children of index TB case household

Despite the initiation of good activities by the NTP, there are still areas where it can work for enhancing the childhood TB cases in the country. Here we propose few areas to improve for the detection and management of childhood TB cases.

## WAY FORWARD

Improvement in underreporting of TB cases, particularly from the private sectors: The number of private sectors has dramatically increased from 1990 to 2016. As with adult TB, the underreporting of childhood TB cannot be undermined. Mandatory notification of childhood TB needs to be in place if we are to increase the number of underreported childhood TB in Nepal.

Improvement in quality of data based on consistent and strict case definition: As TB in children is often paucibacillary, it is quite difficult to diagnose in children. This has resulted in classifying TB based on various inconsistent criteria. This requires adequate monitoring at the national, provincial and local levels. With Nepal having its own National Childhood TB Management Guideline in 2018, this will help standardize diagnosis and treatment. However, a strong monitoring mechanism is still required. Great emphasis should now be given to the quality of TB notification data along with coverage of proper reporting from the country.

Improvement in case-finding strategies: The case-finding strategies have largely been focused on the passive case-finding strategies. While this strategy has been a mainstay in the notification of childhood TB, evidence within Nepal suggests proper household contact screening along with good public-private mix activities resulted in the increased notification of childhood TB. The study conducted by Joshi B et al. showed that screening childhood contacts of smear-positive pulmonary TB index case using a standard screening form by volunteers along with a refund of diagnosis cost to the private sector for a referral to the NTP for treatment showed a significant increase in childhood TB cases.^7^ Although the study was a part of the TB reach project, a detailed work out on the cost-effective analysis and how interventions like these would provide a value of money have not been extensively studied. Currently, the childhood TB guideline focuses on TB screening among malnourished children.

Improvement in reporting format for Health Management Information System: One of the key issues seen with Health Management and Information System data is the difficulty in the analysis of the data to make a meaningful interpretation. While the number of cases and sex affected is easily disaggregated, the sputum conversion and treatment outcome by age is not present. This can be meaningful information for targeted intervention for a particular age group.

Increased capacity of the primary health care network: With Nepal having a robust primary health care network of the health post, urban health clinics, and primary health care centers, we need to capitalize on the health workers in these networks to identify the different signs and symptoms of childhood TB. This is even more important as childhood TB is mostly extrapulmonary.^8^

Investing in the operational and implementation research: A lot of activities have been implemented by the National TB control program recently. It is now time to invest in the research of how things have benefitted with the implementation of the program at the national, provincial, and local levels. There is also a need to strengthen the evidence base on childhood TB with a focus on integration for it with routine health care services
